# A Plea for Higher Medical Education in the South

**Published:** 1894-11

**Authors:** Wm. Simpson Elkin

**Affiliations:** Atlanta, Ga., Professor of Operative Surgery, Southern Medical College; Surgeon to Grady Hospital


### A PLEA FOR HIGHER MEDICAL EDUCATION IN THE SOUTH.*

BY WM. SIMPSON ELKIN, M.D., ATLANTA, GA.,
Professor of Operative Surgery, Southern Medical College; Surgeon to Grady Hospital.

My remarks this morning will be upon the subject of medical education, or rather "A Plea for Higher Medical Education in the South."

There is no disguising the fact that our medical colleges in the South have not been quick to emulate the examples of some of our Northern colleges in their efforts to elevate the standard of medical education; but this fact is not altogether due to any lack of desire on our part, but to some extent to our impecunious condition and inability to secure private, state, or municipal aid to endow and properly equip our medical colleges.

I claim, gentlemen, that to elevate the standard of medical education three requirements, at least, are essential. First: A higher standard of classical education and preliminary training. Second: A graded medical course of, at least, three years. Third: State Medical Examining Boards. If you will think for a moment, young gentlemen, and fully realize the great responsibility that rests on you, you will see in an instant the importance and actual necessity of becoming proficient in every branch of medical science that pertains to your profession. You are intrusted with the lives of human beings; you treat the physical body, a complicated anatomical structure that takes many years of assiduous labor to thoroughly understand, and a slight mistake on your part would be culpable neglect, probably costing your patient's life, and bring into disrepute the profession you are supposed to represent.

*Abstract of opening address delivered at Southern Medical College, October 2, 1894.

Medicine is a grand and noble science which relates to the cure, prevention, and alleviation of disease, a complete knowledge of which requires, in addition to common sense, a mind sufficiently educated or trained to quickly comprehend the technical terms and phrases, and by analyzing the derivation of the words, enable you to discern their meaning. To do this a certain amount of classical learning is absolutely essential, and the young man who has the best qualifications in this direction, with equally as good mind, will more readily learn this science. You, as students who have not this fundamental knowledge, will have to combine with your medical course academic studies, and perform double duty if you keep pace with your classmates, and secure, in the end, your diploma.

In the language of Dr. Pepper, "It avails nothing to point to the rare illustrious men who have leaped at a few bounds from the ploughshare to the front rank of scientific or judicial eminence, and who have served immemorially as instances, to support the fallacy of those who, from selfish motives or from ignorant prejudice, decry thorough education. Let not the rest of us only ordinary sons of toil be mislead by the hope of such exceptional careers."

Your preliminary education should begin before entering upon the study of medicine; but if you have not already obtained the degree of Bachelor of Arts from some college or university, or attended at least a three years' course at an academy or high school of good standing, you should now acquire a thorough knowledge of arithmetic, spelling, elementary English, history of the United States, English composition, and physics. This is the foundation stone that must be laid before you can properly build your superstructure, and in securing this knowledge it is time and money well invested. As the success of one's business depends upon a preparatory knowledge of all the details that relate to that special undertaking, so the professional achievements obtained in the practice of medicine or surgery are contingent upon the preliminary qualifications of the physician or surgeon. As a rule the best educated young men make the best physicians, who rise more rapidly to greater eminence in our profession, and if they are honest, sober, moral, and industrious, are always ready and equipped to sustain the honor and dignity of our profession. Ignorance and illiteracy in our profession are the parents of quackery, and malpractice, and the exponents of all the evils that infest our noble calling.

It is a sad commentary on our profession to note that many students have attended our colleges in years gone by, and are applying now for admittance, who have very little education, and in many instances none whatever. Yet these same young men propose to learn in six or eight months all about the science of medicine, and be legally licensed by our colleges to practice their profession, and kill by their illiteracy and incompetency thousands of human beings. I believe if we could obtain the statistics of lives lost by the culpable mistakes of these wolves in sheep-skin clothing for the past quarter of a century, the number would greatly exceed those killed during the late war between the States.

For five years, from 1888 to 1892 inclusive, before any preliminary entrance examination was required by the medical colleges in Maryland, Kentucky, Tennessee, and Georgia, 5,829 doctors were graduated, and the average percentage of graduates to matriculates in these States was $37\frac{12}{100}$; while for the same length of time the College of Physicians and Surgeons in New York and the Medical Department of the University of Pennsylvania showed an average percentage of graduates to matriculates to be $23\frac{75}{100}$, notwithstanding these institutions had a graded course of three years of nine months each, and required that each student should either have the degree of Bachelor of Arts, or pass a satisfactory preliminary examination in elementary English and Latin. These figures illustrate conclusively, to any sensible mind, the loose and lax manner in which we graduate medical students in the South. I not only regret to say that these four States show the greatest average percentage of graduates to matriculates, but am sorry to confess that Georgia heads the list with an average of $41\frac{91}{100}$; Kentucky, my native State, comes next with $38\frac{89}{100}$; and Maryland and Tennessee close behind in the order named.

Now understand me, gentlemen, for I speak plainly, I mean to say that the four medical colleges in Georgia for the past five years have legally licensed more young men to practice medicine in proportion to the number of matriculates, than the schools of any other

State in the United States or Canada. The 5,829 young men who graduated from the schools of Kentucky, Tennessee, Maryland, and Georgia were not required to stand a preliminary examination before matriculating, and were compelled theoretically to attend only two courses of medicine of five months each, and be allowed by the charter of the institution and laws of the State to lose one-fourth of that time. Any person who could raise $200.00 could leave his plow handles, workshop, or store in the fall, and by paying this amount, or probably less, to the dean of these respective institutions, practically buy a diploma in six or eight months. I estimate that out of these 5,829 graduates, at least 1,700, or thirty per cent. of them are unqualified and unfit to practice medicine, and would be unable to secure a license from any of the State Medical Examining Boards of this country.

Think of four States alone turning out 1,700 ill-trained physicians who are utterly unqualified to practice their profession, but are legalized by these colleges to do so. It seems hard that those young men who have not had the money or opportunity to obtain a good English education, and have been admitted to the lectures without a preliminary examination, should, at the end of two or three years, be refused a diploma; but it would be a greater hardship and misfortune for poor humanity to have turned loose such men in a civilized community, and allow them legally to execute the members of our families.

What I have said in reference to the lax manner in which colleges in the South have matriculated and graduated students in the past five years obtains equally as well with the matriculates of the institutions in the North and West, where there is still as much room and need for a thorough and higher standard of medical education.

Heretofore young men have been allured into adopting our profession as their choice on account of its cheapness and the short length of time required in obtaining a diploma. They look upon it as a trade or business in which they can invest a small amount of money and get quick returns. They have no taste or love for the science, and the interest manifested is purely pecuniary, eight or ten per cent. on the capital involved. If the medical colleges of this country will exact of all persons who contemplate studying medicine the prerequisite of a good English education and an elementary knowledge of Latin, and enforce strictly these requirements in their preliminary examinations, they would improve the quality of their men and raise the standard of medical education; they would cease to be "colleges for revenue only," and the character of our profession would soon be very materially benefited, and the ignorant doctors and unconscionable quack, two dangerous members of our calling, would soon be numbered with things of the past.

I do not censure the students for the part they play in this matter of medical education, for it is human nature for men to obtain things in the easiest way possible and for the least amount of time and money. The medical colleges alone in this country are responsible for this condition by the low standard of medical requirements and the very lax manner of enforcing the preliminary examinations. Let us who favor higher education, both English and medical, present the opportunities, and good results will follow.

In 1887 the Medical Department of the University of Pennsylvania, and, I think, Harvard, were the first schools in this country to adopt the three years' course and require preliminary examinations. These two schools have continued gradually to increase the length of their course, and have now adopted a four years' graded course of nine or ten months each.

One year ago all the medical colleges in the United States and Canada adopted a three years' graded course with only one or two exceptions. Never in the history of our profession has so much been done to improve the standard as in the past ten years.

What we need in the South are fewer medical colleges, more thoroughly equipped laboratories, better clinical advantages, and more endowed institutions. These conditions will be fully realized in the course of a few years, for no section of country is more prosperous or has a brighter future to-day than the South, and as her people grow rich every vocation of life will receive its share of reward, and at no distant day our educational institutions will rival those in the North and the old country.

The science of medicine is a delicate and intricate one, requiring long years of study and practical training, hence making it absolutely necessary that each special branch that relates to it be separated and carefully studied. Physiology, anatomy, and chemistry cannot be thoroughly understood from hearing the didactic lectures; the various functions of the living organism, the science that relates to the organic and inorganic elements of life, and the art of dissecting can only be correctly taught and learned in the laboratories and dissecting rooms.

Every college should afford ample time and opportunity for work in chemical, histological, physiological, bacteriological, and pathological laboratories, for herein lies the fundamental principles upon which you are to build your medical education. Nearly every disease has its own specific germ or micro-organism, and your experimental work in these laboratories will enable you to isolate, culture, and separately study the nature and character of each. By this work you may imbibe enough of the spirit of original research to stimulate you to carry on this work in after years and perhaps be the discoverers of the cause and effect of some diseases yet unknown. You may do for the science of medicine what Lister, Koch, Pasteur, and others in late years have done, and thereby make a name for yourself more enduring than marble and lasting than brass.

It takes time and practice to familiarize yourself with the normal action of the circulatory, respiratory, and other organs of the human body, and still more time and patience for you to be able to discern when these organs are diseased and the exact character of the pathological condition. It requires time and constant practice to become skilled in the use of the microscope, ophthalmoscope, stethoscope, and various other instruments that are employed in the treatment and diagnosis of diseases. The study of disease itself, with its multiplicity of symptoms, varied phenomena, and peculiar complications, necessitates a long and continued attendance upon the clinics, and constant contact with patients in the wards of the hospital. Your work here will be so systematized that the first and second course students will devote most of their time to laboratory work and didactic lectures, and the third course men to practical instructions afforded at the clinics and in the hospital. You must see patients, handle them, make your own diagnoses, if you ever become a proficient diagnostician and successful practitioner. This cannot be done in two years of five months each, but may be done in three years of six months each if you will improve every minute of your time, and take advantage of the opportunities afforded you.

To demonstrate to you what effect the three years' graded course has had in elevating the standard of medicine, I will give you some statistics that Dr. Wm. Pepper has obtained in regard to the degree of success the graduates of the Medical Department of the University of Pennsylvania have made for the past ten years after adopting the three years' course. He says, "no less gratifying is it to learn that no fewer than 600 out of the total of 1,834 graduates have secured appointments as resident physicians, a large majority of the positions being won in competitive examinations; forty-four have already been elected to professorships in medical schools; twenty-nine are lecturers, and forty-eight are assistants. Now, when we reflect upon the large proportion who settled in small towns or rural districts, and also consider how recent is the period I have chosen for inquiry, you will agree with me as to the astonishingly large figures I have quoted." What is true with this institution is equally true with others who had the courage at that time to advance the standard and lengthen the course of medical training.

Having expressed my opinion in regard to the necessity of a good classical education before beginning the study of medicine, and the great importance of a three years' graded course of six months each, I will now give you my reasons for advocating the enactment of State laws, and establishing State Medical Examining Boards, and endeavor to show that this kind of legislation has a good effect upon the character of our profession and tends to elevate its standard. There is no disguising the fact that the majority of colleges in this country are money-making institutions, and have more regard for the *quantity* than the *quality* of the students, and as long as this condition of affairs exists it is the sworn and solemn duty of our lawmakers to pass laws to protect the citizens and aid the victims of these ignorant doctors.

Medical diplomas are often articles of merchandise that cost $1.00 and are sold for $30.00, hence are no index to a man's qualifications or fitness to practice medicine. Every quack whose picture adorns the pages of our daily and weekly papers has a diploma, yet he is an unscrupulous charlatan who lives by humbugging and robbing innocent people. The criminal abortionist has a diploma, yet he is an assassin and a murderer. If for no other reason than to rid the profession of these two classes of doctors, State legislation is indicated, and I am pleased to say has been enacted in almost all our States. In Alabama, Arkansas, Florida, Kentucky, New Jersey, New York, North Carolina, South Carolina, North Dakota, Oregon, Pennsylvania, South Dakota, Texas, Utah, Virginia, Tennessee, Washington, Missouri, West Virginia, Montana, New Mexico, Delaware, Colorado, California, Louisiana, Minnesota, and Nebraska, the diploma either confers no right to practice, or certificates are issued upon the diplomas of colleges of good standing after examinations by State Examining Boards. By colleges in good standing are meant those schools that teach a three years' graded course. In Georgia, Arizona, Idaho, Indiana, Kansas, Michigan, Nevada, Ohio, Oklahoma, Vermont, Wyoming, and the District of Columbia, the simple registration of a diploma is all that is necessary, while in Rhode Island, New Hampshire, Massachusetts, and Maine, there are no legal requirements. I have every assurance that this State, and others in the same category, will, within a few months, enact laws governing the right to practice medicine.

I have had occasion reeently to inquire into the effect of the workings of these boards in the different States, and have yet to receive any but the most favorable and gratifying accounts. From California to Florida, from Washington to New York, the reports indicate marked improvement in the general character of the medical profession.

Dr. W. W. Bullard, Secretary of Montana Board of Medical Examiners, says: The work of our board has been remarkably successful. When we started to enforce the law in 1889 the Territory was overrun with quacks and all sorts of illegal practitioners. At the present time I do not know of more than two or three without certificates from this board, and we are after them. We require in every case an affidavit showing that the applicant has attended not less than three full courses of not less than four months each, and present a diploma from a recognized college and pass an examination before the board. The requirements will be raised to four courses of six months each. The work of the board has had a marked effect upon the personnel of the profession of the State. In a word our law is a success.

Dr. James Brown, Secretary Oregon Board of Medical Examiners, says: Number of examinations, 204; of these 116 received license, and eighty-eight, or forty-three per cent., were not deemed worthy of license. As to the effect of the working of the board, I think I may claim—

1. That under the operation of the medical law charlatanism and quackery have been reduced to a minimum.

2. That the standard of medical education has been elevated, the two medical colleges of this city having adopted the requirements insisted on by this board.

3. That the influence of Oregon's medical laws has been felt outside the limits of the State. The rejection, on supplementary examination, of persons holding diplomas from colleges not deemed by this board to be in good standing, has reacted on the colleges and stimulated them to make their requirements stricter and their standard higher.

Dr. M. J. Lewi, Secretary of New York State Examining Board, says: The general personnel of the medical profession is improving perceptibly, and necessarily so. Our law went into effect September 1, 1894, and since that time all who desire to practice medicine in New York State (with the exception of those who had matriculated in our State previous to that date), no matter where graduated, must satisfy the following requirements before being admitted to our examination for license:

1. More than twenty-one years of age.

2. Good moral character.

3. Preliminary academic education, minimum requirements three years study at a registered (first-class) high school.

4. At least three full courses of lectures at a medical college of satisfactory standard, no two courses in one year.

5. Full degree of doctor or bachelor of medicine.

These requirements debar from our examinations many who would otherwise be knocking at our doors for admission, and consequently our applicants are better fitted to enter on their life-work than heretofore.

Dr. Jerome Cochran, Senior Censor Medical Association, State of Alabama, says: Our law is rapidly revolutionizing the medical profession of the State. Already the improvement has been immense. We can't fall below the college standard because we don't examine any but graduates, and we, in fact, rise above the college standard, because we reject twelve to fifteen per cent. of the graduates examined.

Dr. Thomas McDavitt, Secretary Minnesota State Examining Board, says: There have been four hundred and eighty different applicants examined, and eighty-one of them have been refused license. The effect of our law upon the personnel of the physicians has been admirable, and I think the physicians of Minnesota will compare more than favorably with any other State in the Union in all respects.

Dr. J. Hall, Secretary Colorado State Examining Board, says: The law has had a great effect in improving the personnel of the profession in the State. About thirty per cent. of those taking the examination have failed during the past five years.

I could quote similar extracts from the letters of secretaries of examining boards of many other States, but this is sufficient to show the good effect the workings of these boards have upon the general character of the medical profession. They tend also to elevate this standard by requiring the colleges to teach a three years' graded course, and by refusing to issue license to physicians who are incompetent to practice medicine.

Since it might be interesting, for you know how utterly unqualified the graduates of some of our colleges are, I will give you a few questions and answers as reported by one or two of these examining boards.

In Virginia the following are some of the questions and answers:

"Give general and descriptive anatomy of the stomach." "It is the organ where food is digested; it is a very extensive organ."

"Describe or define a cell." "It is a place of confinement."

"The normal temperature of the human body is from 112 to 140, and the average respirations are 70 per minute."

"The technical name of rhubarb is columbo." "The dose of antipyrin for a child five years old is 15 grains every three hours, and that of morphia hyperdermically for a child of the same age would be ¼ of a grain, and if that doesn't give relief I would give ½ grain.

To the question of the diagnosis of the dislocation of the head of the femur on the dorsum of the ilium, it is replied, "Don't know much about the diagnosis, but the treatment is amputation."

"The symptoms of œdema of the glottis are that the patient feels husky and has a sore throat. I would amputate it if necessary. I would do the operation within three or four months if it was a bad case."

"Extra-Uterine pregnancy may be a fungoid growth or tumor, fibroid in its character, or any extra growth in the utrous would be called extra-uterine pregnancy."

"The best way to facilitate the expulsion of the placenta is to let the woman get up and walk about the room, always allowing five minutes to elapse after delivery before requiring her to get up and walk."

Following are some Minnesota questions and answers:

"The scrofulous diathesis is known by a peculiar greasy exudation from the axilla or inside of the thigh, possibly behind the ears; has a sour, fetid, strong smelling odor."

"Symptoms of cardiac dilatation—dull pain at pit of stomach, and a feeling of water in the bowels, ematiation, anema, loss of flesh. Treatment, put patient on a milk diet, and give rectal onema of pepsonical food, and a nerve tonic to tone up the system."

"Locomotor ataxia.—Hear all the lesions or pathology changes is situated in the fourth ventricle of the brain, and a slight pathological change in the penducles of the serebellum (I am rattled if that ain't right)."

"Placenta prævia.—This is a retaining of the placenta structures after the delivery of child, and a part of the placenta, all is to be done in this case is to introduce the hand or an instrument and remove any of the membranes that is left or curet the utris."

I am sure that this is sufficient argument to convince any one that a medical diploma is not a guarantee of one's fitness to practice medicine; hence it becomes the imperative duty of the different States to enact laws admitting only physicians of acknowledged merit to exercise the right to pursue their profession. These laws would also serve as a public blessing, in that they protect our citizens from the malpractice of ignorant doctors, and the humbuggery of quacks.

